# Characteristics and Cost of Unscheduled Hospitalizations in Patients Treated with New Oral Anticancer Drugs in Germany: Evidence from the Randomized AMBORA Trial

**DOI:** 10.3390/jcm11216392

**Published:** 2022-10-28

**Authors:** Pauline Dürr, Florian Meier, Katja Schlichtig, Anja Schramm, Lukas Schötz, Martin F. Fromm, Frank Dörje

**Affiliations:** 1 Pharmacy Department, Erlangen University Hospital, 91054 Erlangen, Germany; 2 Institute of Experimental and Clinical Pharmacology and Toxicology, Friedrich-Alexander-Universität Erlangen-Nürnberg, 91054 Erlangen, Germany; 3 Comprehensive Cancer Center Erlangen-EMN, Erlangen University Hospital, 91054 Erlangen, Germany; 4 Department of Management and Economics, SRH Wilhelm Löhe University of Applied Sciences, 90763 Fürth, Germany; 5 AOK Bayern, 93055 Regensburg, Germany

**Keywords:** drug-related problems, hospital admissions, hospitalization cost, intensified pharmacological/pharmaceutical care, oral anticancer treatment, potential savings, adverse drug reactions

## Abstract

Drug-related problems (e.g., adverse drug reactions, ADR) are serious safety issues in patients treated with oral anticancer therapeutics (OAT). The previously published randomized AMBORA trial showed that an intensified clinical pharmacological/pharmaceutical care program within the first 12 weeks of treatment reduces the number and severity of ADR as well as hospitalization rates in 202 patients. The present investigation focused on unscheduled hospitalizations detected within AMBORA and analyzed the characteristics (e.g., frequency, involved OAT) and cost of each hospital stay. To estimate the potential savings of an intensified care program in a larger group, the absolute risk for OAT-related hospitalizations was extrapolated to all insureds of a leading German statutory health insurance company (AOK Bayern). Within 12 weeks, 45 of 202 patients were hospitalized. 50% of all unscheduled hospital admissions were OAT-related (20 of 40) and occurred in 18 patients. The mean cost per inpatient stay was EUR 5873. The intensified AMBORA care program reduced the patients’ absolute risk for OAT-related hospitalization by 11.36%. If this care program would have been implemented in the AOK Bayern collective (3,862,017 insureds) it has the potential to reduce hospitalization rates and thereby cost by a maximum of EUR 4.745 million within 12 weeks after therapy initiation.

## 1. Introduction

During the last two decades, new oral anticancer drugs (e.g., kinase inhibitors) are increasingly used for cancer treatment [1]. The application mode is more convenient compared to intravenously administered cytotoxic drugs and allows outpatient treatment in the majority of cases. Nevertheless, oral anticancer treatment is not a harmless version of chemotherapy [1]. Oral anticancer drugs are associated with a high risk of potential patient harm due to drug-related problems (adverse drug reactions (ADR) and medication errors) [2,3,4,5,6]. The spectrum of ADR caused by oral anticancer drugs ranges from usually mild, self-limiting incidents such as abdominal pain to severe events such as neutropenia or infections, which can lead to hospital admissions or even death [4,6].

Several previous studies analyzed anticancer treatment-related hospitalizations (e.g., regarding frequency, underlying ADR, or associated cost) [7,8,9,10,11,12,13,14,15,16,17,18,19,20,21,22]. However, the characteristics of those reported hospital admissions do not apply to the broad range of new oral anticancer drugs. Most of the reporting trials only focused on patients receiving intravenous chemotherapy [8,9,11,18,19,20,21,22], included only a small number of patients treated with oral anticancer drugs [7,10,12,13], or took only account of specific tumor entities [14,15,16,17]. The high economic impact of treatment-related hospitalizations is obvious [9,11,12,14,15,16,17,18,19] and should be addressed to reduce healthcare costs.

Clinical care programs for patients receiving oral anticancer drugs can reduce hospitalizations and have thereby the potential for cost savings, but data is very limited up to now [12].

The prospective, randomized, multicenter trial AMBORA (Medication Safety With Oral Antitumor Drugs) showed a reduction of ADR and treatment-related hospitalization rates when an intensified care program is additionally applied to the standard of care in patients with new oral antitumor therapy [6]. In this trial, clinical pharmacologists/pharmacists performed structured management of adverse drug reactions including prophylactic treatment (e.g., skin care), education of patients in self-management, close symptom monitoring, and early interventions [6].

Based on the data of the AMBORA trial, additional post hoc analyses were performed. The aims of the present investigation were the following: First, to analyze the characteristics of treatment-related hospital admissions (e.g., incidence, associated drug-related problems, involved oral anticancer drugs). Second, to assess the cost caused by those hospitalizations. Additionally, third, to estimate the potential savings of an intensified care program as provided in the AMBORA trial [6] extrapolated to the largest Bavarian statutory health insurance (SHI) company AOK Bayern, using real-world prescription data.

## 2. Materials and Methods

### 2.1. Study Design and Patients

For the present work, we performed additional analysis of the previously published prospective, randomized, multicenter AMBORA trial [5,6,23]. Patients newly started on new oral anticancer drugs (approval date after capecitabine in 2001 in Germany) were randomly assigned to receive standard of care (control group) or an additional, intensified clinical pharmacological/pharmaceutical care program on top (intervention group) over 12 weeks. The care program included, e.g., medication management and structured patient counseling (e.g., regarding prevention and treatment of ADR). Patients were recruited between 15 November 2017 and 28 January 2020 (27 months). The trial was registered at the German Clinical Trials Register (DRKS00013271) and approved by the Ethics Committee of the Friedrich-Alexander-Universität Erlangen-Nürnberg. Written informed consent was obtained from all patients prior to study entry.

### 2.2. Data Collection

Demographic characteristics and clinical data were collected in structured patient interviews at baseline and updated after 4 and 12 weeks, always confirmed and completed with the physicians’ documentation in the medical records. Consistent data assessment and documentation were ensured by using standard operating procedures, documentation forms, and checklists [6].

### 2.3. Assessment of Drug-Related Problems

Adverse drug reactions and medication errors were assessed patient-reported during structured patient interviews, and objective ADR (e.g., laboratory values) were extracted from the medical record. The Common Terminology Criteria for Adverse Events (CTCAE version 4.03) was used to grade the severity of ADR [24]. To minimize bias, the causality assessment of all ADR was conducted according to the World Health Organization Uppsala Monitoring Centre (WHO-UMC) system [25]. Only adverse drug reactions scored as ‘possible’, ‘probable/likely’, or ‘certain’ were categorized as ‘related to the oral anticancer treatment (OAT)’ and thereby included in the analysis.

### 2.4. Assessment of Hospitalizations

During 12 weeks of follow-up, all hospitalizations were systematically assessed during patient interviews and aligned with the medical documentation (e.g., physician’s letters and medical health records). If any information was dissenting or missing (e.g., hospitalization in another hospital than Erlangen University Hospital), the responsible physician was contacted for information and clarification. We assessed the hospital location, date of admission, length of stay, underlying reasons for hospitalization, and whether it was scheduled or unscheduled. In the case of unscheduled hospitalizations, we assessed if the hospital admission was caused by OAT-related adverse drug reactions according to the WHO-UMC system [25]. In the following, these unscheduled hospitalizations related to oral anticancer treatment are named ‘OAT-related hospitalizations’.

### 2.5. Economic Data Collection and Analysis

For the economic analysis, hospital inpatient data were collected. This standardized data set was defined in section 21 of the Hospital Remuneration Act (KHEntgG) and contains the German Diagnosis Related Groups (G-DRG) cost for an inpatient stay. The cost reported in this analysis was calculated on the same basis used by German statutory health insurance (SHI) companies. Outpatient healthcare costs were not addressed in this analysis.

### 2.6. Data Analysis SHI Collective (AOK Bayern)

The data utilization from the SHI collective of AOK Bayern was approved by the responsible regulatory authority (Bavarian State Ministry of Health and Care). Adult patients (≥18 years) with first outpatient prescription of new oral anticancer drugs (approval date after capecitabine in 2001 in Germany consistent with the AMBORA trial [6] protocol) within the AMBORA recruitment period of 27 months (15 November 2017 to 28 January 2020), were included. Patients were only considered in the analysis if they were insured by AOK Bayern for at least 95% of the time period. If there was no prescription of the same oral anticancer drug one year before a prescription date, this was defined as ‘first prescription’.

The following data were provided by AOK Bayern: the number of patients started on new oral anticancer drugs and the number of unscheduled hospital admissions (documented as ‘emergency hospitalizations’) in those patients within 12 weeks after first prescription. Moreover, AOK Bayern reported the number of adult (≥18 years) insureds on 31 December 2018 and 31 December 2019. The mean of both years was defined as the mean number of insureds. Using this dataset, further descriptive analyses were performed (e.g., hospitalization rates and involved oral anticancer drugs).

### 2.7. Estimating the Potential Savings of Direct Hospital Cost by an Intensified Care Program as Provided in AMBORA

We first calculated the absolute risk for OAT-related hospitalization within 12 weeks after the start of a new oral anticancer drug for both, the intervention and the control group in AMBORA. Subsequently, we estimated the incidence of hospitalizations for the SHI collective of AOK Bayern for two scenarios: All patients with a new oral anticancer drug therapy within this collective would receive (1) standard of care (such as AMBORA control group), or (2) an intensified clinical pharmacological/pharmaceutical care program (such as AMBORA intervention group). The basis for this calculation was the number of patients newly started on new oral anticancer drugs in the insureds of AOK Bayern.

### 2.8. Statistical Analysis

For data storage, data preparation, and statistical analysis, we used Microsoft Access and SPSS 20 (IBM SPSS Statistics for Windows, version 20.0, IBM Corporation, Armonk, NY, USA). Results are presented with mean, standard deviation (±SD), median with interquartile range (IQR), and range. The comparison between the two study groups was performed using the student’s *t*-test. Since cost data usually have a non-normal distribution with right-skewness, we, therefore, used non-parametric bootstrap techniques (10,000 replications) to handle uncertainties and calculated the bias-corrected and accelerated 95% confidence interval (BCa 95% CI) [26,27,28].

## 3. Results

### 3.1. Patients within AMBORA

**Figure 1:**
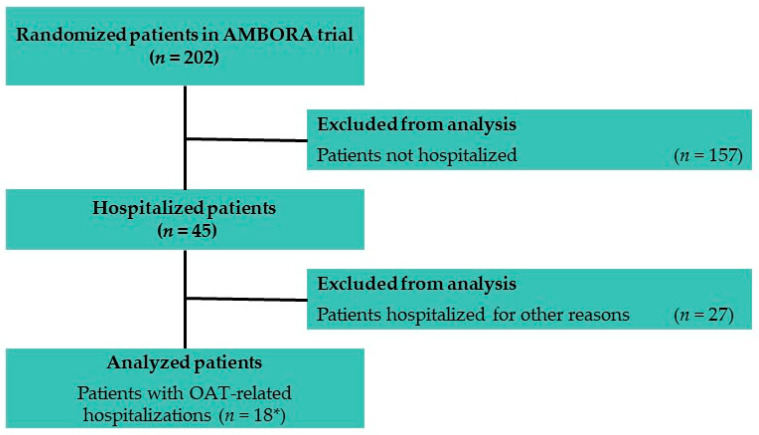
Flowchart for selection of the analyzed patients with OAT-related hospitalizations within the AMBORA trial. * Two patients were hospitalized twice within 12 weeks. Abbreviations: OAT, oral anticancer treatment.

The flowchart according to CONSORT and the baseline characteristics of the 202 patients enrolled in the AMBORA trial have previously been published [6]. Figure 1 shows the selection of hospitalized and analyzed patients within the AMBORA collective. The baseline characteristics of the 18 patients with OAT-related hospitalizations are shown in Table 1.

#### 3.1.1. Characteristics of Hospitalizations within AMBORA

**Figure 2:**
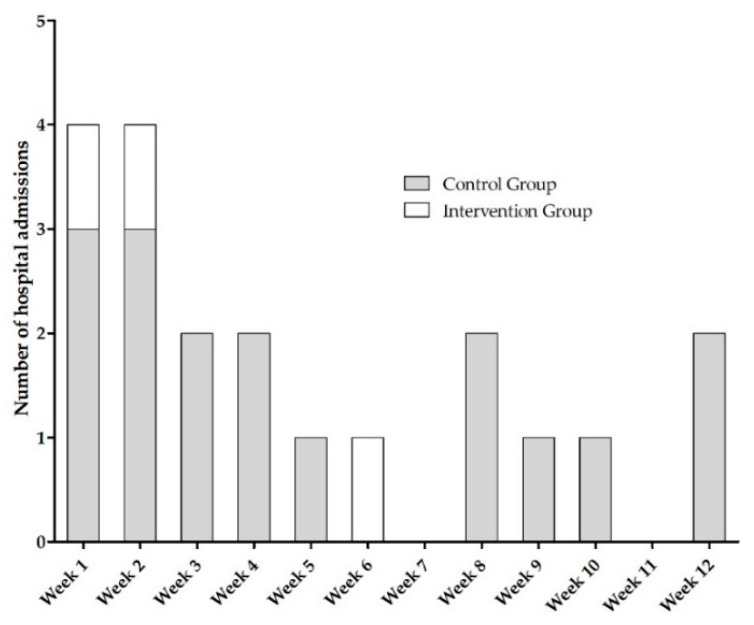
Number of OAT-related hospitalizations and their date of occurrence within AMBORA. Abbreviations: OAT, oral anticancer treatment.

60 cases of hospitalizations were documented, thereof 20 scheduled hospital admissions. Of the remaining 40 unscheduled hospitalizations, 20 incidents (50%) were OAT-related. These 20 hospital admissions occurred in 18 patients (3 intervention group, 15 control group, *p* < 0.004 [6], Figure 1). Figure 2 shows the date of occurrence of OAT-related hospital admissions during the 12 week follow-up. The majority of hospitalizations (70%) occurred within the first 6 weeks of treatment.

#### 3.1.2. Oral Anticancer Drugs Associated with Hospitalizations in AMBORA

**Figure 3:**
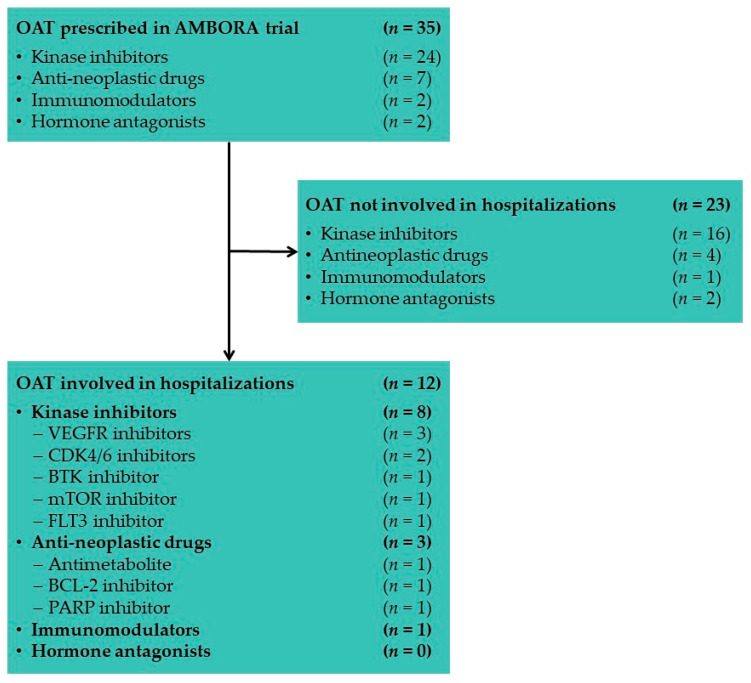
Overview of OAT prescribed and involved in OAT-related hospitalizations including their underlying mechanism of action and target structures within AMBORA. Abbreviations: BCL-2, B-cell lymphoma protein 2; BTK, Bruton’s tyrosine kinase; CDK4/6, cyclin-dependent protein kinases 4/6; FLT3, FMS-like tyrosine kinase; mTOR, mitogen-activated protein kinase; OAT, oral anticancer treatment; PARP, Poly(ADP-ribose) polymerase; VEGFR, vascular endothelial growth factor receptor.

As shown in Figure 3, the 202 patients randomized in AMBORA were treated with 35 different oral anticancer drugs, predominantly kinase inhibitors. Of the 24 different kinase inhibitors included in AMBORA, eight were related to unscheduled hospitalizations. Inhibitors of VEGFR (vascular endothelial growth factor receptor) and CDK4/6 (cyclin-depending kinases 4 and 6) were most frequently involved in hospital admissions.

#### 3.1.3. Adverse Drug Reactions Associated with Hospitalizations in AMBORA

**Figure 4:**
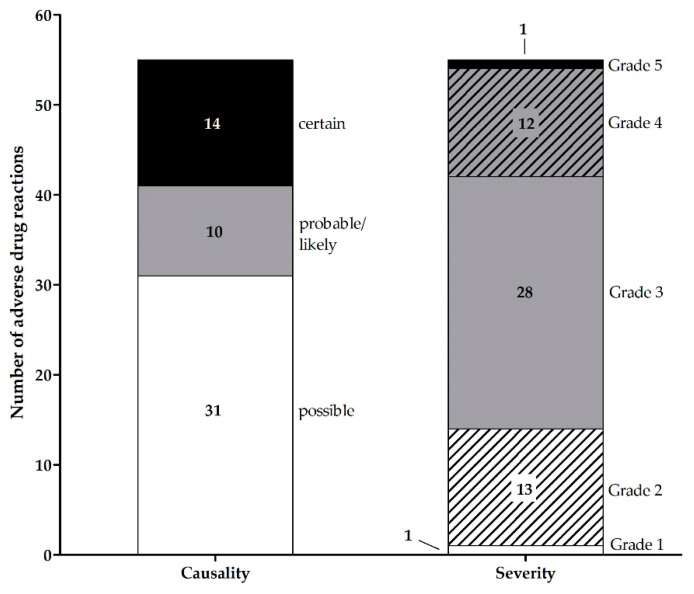
Number of OAT-related adverse drug reactions associated with hospitalizations, stratified for causality (according to WHO-UMC) and severity (according to CTCAE) within AMBORA. Abbreviations: CTCAE, Common Terminology Criteria for Adverse Events; OAT, oral anticancer treatment; WHO-UMC, World Health Organization Uppsala Monitoring Centre system.

Table 2 shows the 55 different ADR that were related to hospital admissions in the 18 analyzed patients. As shown in Table 2, blood count and gastrointestinal disorders were the most common types of ADR. In 70% of all hospital admissions, a combination of ADR (median: 2, range: 1–8) led to hospitalization. The causality (according to WHO-UMC) and severity (according to CTCAE) of all ADR are shown in Figure 4. 75% of ADR that led to hospitalizations were severe (CTCAE grade ≥ 3). One ADR with grade 5 (lethal) occurred. This was a septic complication in a patient with leucopenia related to everolimus. In one case a medication error led to hospitalization. In this case, oral bleeding occurred after a planned tooth extraction during treatment with lenvatinib. This probably could have been avoided by a perioperative treatment interruption.

### 3.2. Characteristics of Hospitalizations within the SHI Collective

Within the AMBORA recruitment period, 8102 first prescriptions of new oral anticancer drugs were observed in 7106 patients within the SHI collective of AOK Bayern. During the first 12 weeks after therapy initiation, 2761 emergency hospitalizations of any reason occurred. Normalizing the number of hospitalizations to the number of prescriptions leads to an overall hospitalization rate of 34.1% within 12 weeks after the first prescription (Table 3). As shown in Table 3 stratified for the mechanism of action and normalized by the number of prescriptions, the lowest rate of emergency hospitalizations was found for BCR-ABL inhibitors (21.6%), the highest for VEGFR inhibitors (50.1%).

### 3.3. Cost of OAT-Related Hospitalizations within AMBORA

Table 4 gives an overview of the 18 hospitalized patients, their oral anticancer treatment, the types of adverse drug reactions associated with the hospitalization, the length of hospital stays, DRGs, and cost per patient for inpatient stay. There were 19 different DRGs with R03Z (Lymphoma and leukemia with a specific OR procedure) as the most expensive DRG (EUR 26,389) and X62Z (Poisoning/Toxic Effects of Drugs, Medicines, and Other Substances) with the lowest cost (EUR 957). The mean cost per patient was EUR 8407 (SD: EUR 6501) in the intervention group, and EUR 5366 (SD: EUR 8014) in the control group, respectively. The difference was not statistically significant (*p* = 0.568). The overall mean was EUR 5873 (SD: EUR 6612; IQR: EUR 4582; Range: EUR 956–EUR 26,389).

The prevention of hospital admissions by applying an intensified clinical pharmacological/pharmaceutical care program led to a reduction in hospitalizations and thereby cost (Table 4). In AMBORA, overall hospitalization costs were EUR 25,223 in the intervention group compared to EUR 80,501 in the control group (Table 4).

According to the AMBORA trial [6], the absolute risk detected for an OAT-related hospitalization in the intervention group was 3.06% and in the control group 14.42% (Table 5).

### 3.4. Potential Savings within the SHI Collective

**Figure 5:**
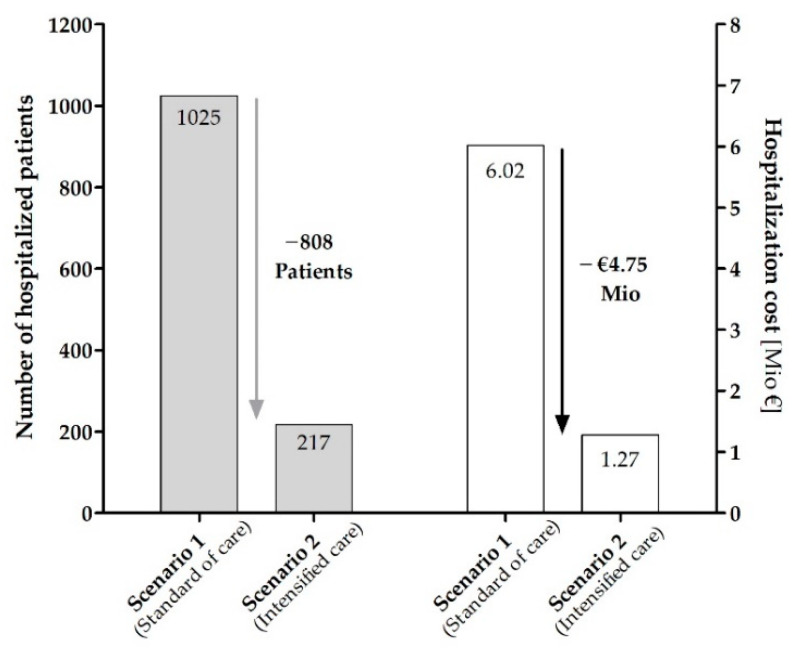
Estimated number of patients with OAT-related hospitalizations and potential savings based on the scenario analysis within the SHI collective of AOK Bayern. Abbreviations: Mio, million; OAT, oral anticancer treatment; SHI, statutory health insurance.

Within the SHI collective of AOK Bayern (3,862,017 insureds), we identified 7106 patients who started treatment with a new oral anticancer drug within the AMBORA recruiting time period (27 months). The scenario analysis was based on the absolute risk for OAT-related hospitalization evaluated in AMBORA (3.06 vs. 14.42%, Table 5) and the real-world prescription data from AOK Bayern. This led to an estimated reduction of 808 patients with OAT-related hospitalization within 12 weeks after the first prescription of new oral anticancer drugs during the AMBORA recruitment period of 27 months (1025 hospitalized patients with standard care vs. 217 hospitalized patients with intensified clinical pharmacological/pharmaceutical care, Table 5). This may reduce the hospitalization cost by a maximum of EUR 4.745 million within the SHI collective of AOK Bayern based on the mean hospital cost of EUR 5873 as detected in AMBORA (Figure 5).

## 4. Discussion

Within recent years, the use of oral anticancer drugs substantially raised. This paradigm change leads to an increasing shift from anticancer drugs intravenously administered under medical observation to oral therapies self-administered by patients at home. The patient’s responsibility for self-management of adverse drug reactions and correct drug intake is significantly higher and requires special attention. In the present work, we performed an additional differentiated analysis of OAT-related hospitalizations, respective adverse drug reactions, and direct hospitalization costs in the German healthcare system.

The AMBORA trial demonstrated that an intensified clinical pharmacological/pharmaceutical care program reduces the number and severity of ADR and leads to a reduction in hospitalizations [6]. The majority of hospital admissions (70%) occurred within the first 6 weeks of oral anticancer therapy (Figure 2). Thus, an intensified patient care program seems to be especially useful after initiation of oral anticancer drugs. VEGFR and CDK4/6 inhibitors were the drug classes most frequently involved in hospitalizations within AMBORA (Figure 3). All new oral anticancer drugs were included in AMBORA thereby leading to small sample sizes per drug. Thus, it is not indicated to draw final conclusions about particularly problem-proned drugs associated with OAT-related hospitalizations. Consistent with the AMBORA data, the drug class most frequently involved in emergency hospitalizations within 12 weeks in the SHI collective of AOK Bayern were VEGFR inhibitors (Table 3). VEGFR inhibitors (e.g., sorafenib, cabozantinib) are the so-called multikinase inhibitors, which address various off-targets and thereby have a wide range of ADR [29]. This leads to a rising risk for OAT-related hospitalizations. The lowest hospitalization rate within the SHI collective was found for BCR-ABL inhibitors (e.g., imatinib). Those drugs are predominantly used for the long-term treatment of Philadelphia chromosome-positive chronic myeloid leukemia (Ph+ CML). Patients with Ph+ CML are younger (median age 57 years) compared to most other cancer types treated with OAT and thereby often have fewer comorbidities [30]. It seems reasonable, that patients treated with BCR-ABL inhibitors have low hospitalization rates. In the analysis of the SHI collective of AOK Bayern, only emergency hospitalizations were included. It has to be mentioned, that there is no possibility to distinguish between OAT-related hospitalizations and hospital admissions due to other reasons (e.g., cancer-related) using hospitalization data extracted from the German healthcare system.

The leading types of ADR associated with hospitalizations in the AMBORA trial were blood count disorders such as neutropenia (34.5%) and gastrointestinal disorders (30.9%) (Table 2). There is little evidence from other trials about hospitalization rates caused by ADR of oral anticancer drugs [7,10,12,13,14]. A former retrospective study by Wong et al. on ambulatory patients who were admitted to a hospital within 30 days after the administration of anticancer treatment, showed that about 19% of hospitalizations were due to treatment-related adverse events [7]. In this trial, the leading adverse events that resulted in hospitalization were gastrointestinal disorders (48%, 26 of 54) followed by infections (26%, 14 of 54) [7]. In the AMBORA trial, 50% of unscheduled hospital admissions were OAT-related. However, in contrast to AMBORA, most patients in the trial of Wong et al. were treated with intravenous cytotoxic drugs or checkpoint inhibitors and only a small proportion received oral anticancer drugs [7]. Moreover, the observation period differed, 30 days instead of 12 weeks. Overall, the findings of other trials are difficult to compare due to the restricted numbers of included entities treated with oral anticancer drugs [10,13,14] or because underlying ADR leading to hospital admission were not reported in detail [12,13].

Matching the types of ADR that led to hospitalizations and the documented DRG codes presented in Table 4, it becomes evident, that most ADR are not well documented or not represented in the DRG coding system. Only in one case, the DRG Code (X62Z–Poisoning/Toxic Effects of Drugs, Medicines and Other Substances) itself implicates that an ADR may be involved in hospitalization. The challenge to assess treatment-related hospitalizations in the G-DRG coding system has been reported in a former study and is a well-known limitation of the G-DRG system [31].

The intensified care program applied in the AMBORA trial led to a significant reduction in hospitalization rates. The absolute risk reduction to have an OAT-related hospitalization was 11.36%, thereby leading to a substantial cost reduction for inpatient stays.

Based on the absolute risk for hospitalization evaluated in AMBORA, we carried out a scenario analysis and extrapolated the randomized trial data to real-world data of the largest Bavarian SHI, AOK Bayern. The SHI collective from AOK Bayern included 7106 patients started on new oral anticancer drugs in the AMBORA recruitment period (27 months). Applying the scenario analysis, a reduction of 808 patients with hospital admissions and a maximum saving potential of EUR 4.745 million could be estimated.

Although it is well-known, that clinical pharmacists/pharmacologists are an important factor in medication safety and patient care, they are still less frequently involved in the medication process in German hospitals compared to other countries (e.g., UK, USA) [32,33]. Especially in patients treated with new oral anticancer drugs, the comprehensive integration of clinical pharmacists/pharmacologists in the treatment team is highly valuable, since the AMBORA trial showed its potential to improve medication safety [6].

However, we are aware of certain limitations. Albeit the core data were assessed in a prospective, randomized, multicenter trial, the absolute number of hospitalized patients is small. Thus, leading to an uncertainty of the absolute risk for unscheduled hospital admissions. The calculation of the potential savings is based on the mean cost of OAT-related hospital stays within AMBORA, which showed high variability. The reimbursement rates for DRGs are based on the Bavarian hospital base rate, which differs from other statewide base rates. DRG data of our analyses were collected mainly for administrative purposes and may not precisely reflect real healthcare costs. Data about the incidence and cost of hospitalizations after the follow-up period of 12 weeks were not evaluated in our analysis. To fully assess the net benefits of the intensified clinical pharmacological/pharmaceutical care program, it would have been necessary to consider the costs of the care program (e.g., staff cost) as well as other savings (e.g., reduced drug wastage due to prevention of treatment discontinuations). Further health economic evaluations considering additional factors (e.g., reduced costs due to prevention of rehabilitation, and the impact of higher quality of life) are necessary for an all-encompassing assessment of the economic outcomes.

## 5. Conclusions

The AMBORA trial data in conjunction with real-world data from the SHI AOK Bayern demonstrate the major economic burden of unscheduled hospitalizations in patients treated with new oral anticancer drugs. The implementation of an intensified clinical pharmacological/pharmaceutical care program has the potential to restrict this burden substantially and reduce patients’ exposure to adverse drug reactions.

## Figures and Tables

**Table 1:** Baseline characteristics of the analyzed patients with OAT-related hospitalizations within AMBORA.

Characteristic	No. (%) Total (*n* = 18)
**Age, years (mean, range)**	67.8 (47–91)
**Female sex**	11 (61.1)
**Cancer type**	
***Solid tumors***	
Breast	3 (16.7)
Soft tissue sarcoma	3 (16.7)
Small intestine	2 (11.1)
Others *	4 (22.2)
***Hematologic malignancies***	
Acute myeloid leukemia	2 (11.1)
Mantle cell lymphoma	2 (11.1)
Multiple myeloma	2 (11.1)
**ECOG performance status**	
0	4 (22.2)
1	11 (61.1)
2	2 (11.1)
3	1 (5.6)
**Number of drugs ^+^ (median, range)**	9 (3–24)
**Oral anticancer drug**	
**Protein kinase inhibitors**	13 (72.2)
Pazopanib	3 (16.7)
Everolimus	2 (11.1)
Ibrutinib	2 (11.1)
Palbociclib	2 (11.1)
Cabozantinib	1 (5.6)
Lenvatinib	1 (5.6)
Midostaurin	1 (5.6)
Ribociclib	1 (5.6)
**Antineoplastic agents**	3 (16.7)
Niraparib	1 (5.6)
Tegafur, gimeracil, oteracil	1 (5.6)
Venetoclax	1 (5.6)
**Immunomodulators**	2 (11.1)
Lenalidomide	2 (11.1)

* Others: cancer types that were included only once (colorectal cancer, hepatocellular carcinoma, ovarian cancer, thyroid cancer). ^+^ Number of active ingredients in approved drugs at baseline. Abbreviations: ECOG, Eastern Cooperative Oncology Group.

**Table 2:** Number of adverse drug reactions associated with OAT-related hospitalizations within AMBORA.

CTCAE Term	No. (%) Total (*n* = 18 Patients)	
	Any Grade	Grade ≥ 3
**Total**	55 (100)	41 (74.5)
**Blood count disorders**	19 (34.5)	19 (46.3)
Lymphocyte count decreased	6 (10.9)	6 (14.6)
Neutrophil count decreased	5 (9.1)	5 (12.2)
White blood cells decreased	5 (9.1)	5 (12.2)
Anemia	2 (3.6)	2 (4.9)
Platelet count decreased	1 (1.8)	1 (2.4)
**Gastrointestinal disorders**	17 (30.9)	5 (12.2)
Nausea	3 (5.5)	1 (2.4)
Anorexia	2 (3.6)	0 (0)
Diarrhea	2 (3.6)	1 (2.4)
Mucositis oral	2 (3.6)	1 (2.4)
Vomiting	2 (3.6)	1 (2.4)
Anal mucositis	1 (1.8)	0 (0)
Bloating	1 (1.8)	0 (0)
Constipation	1 (1.8)	1 (2.4)
Dysgeusia	1 (1.8)	0 (0)
Gastroesophageal reflux disease	1 (1.8)	0 (0)
Laryngeal mucositis	1 (1.8)	0 (0)
**Cardiovascular and lung disorders**	6 (10.9)	6 (14.6)
Dyspnea	2 (3.6)	2 (4.9)
Hypertension	2 (3.6)	2 (4.9)
Pleural effusion	1 (1.8)	1 (2.4)
Pulmonary fibrosis	1 (1.8)	1 (2.4)
**Infections**	5 (9.1)	4 (9.8)
Anorectal infection	1 (1.8)	1 (2.4)
Bronchial infection	1 (1.8)	1 (2.4)
Fever	1 (1.8)	0 (0)
Infections, other	1 (1.8)	1 (2.4)
Sepsis	1 (1.8)	1 (2.4)
**Bleeding disorders**	3 (5.4)	2 (4.9)
Hematoma	1 (1.8)	1 (2.4)
Lower gastrointestinal hemorrhage	1 (1.8)	1 (2.4)
Oral hemorrhage ^#^	1 (1.8)	0 (0)
**Organ failure**	2 (3.6)	2 (4.9)
Heart failure	1 (1.8)	1 (2.4)
Hepatic failure	1 (1.8)	1 (2.4)
**Other adverse drug reactions**	3 (5.5)	3 (7.3)
Fatigue	1 (1.8)	1 (2.4)
Hypokalemia	1 (1.8)	1 (2.4)
Retinal detachment	1 (1.8)	1 (2.4)

^#^ Adverse drug reaction caused by a medication error. Abbreviations: CTCAE, Common Terminology Criteria for Adverse Events.

**Table 3:** Numbers and rates of emergency hospitalizations within 12 weeks after first prescription of new oral anticancer drugs within the SHI collective of AOK Bayern.

OAT *	No.		Hospitalization Rate, Normalized by Prescription Numbers (%)
	First Prescriptions	Emergency Hospitalizations	
	Total (*n* = 8102)	Total (*n* = 2761)	34.1
**KINASE INHIBITORS**	4333	1536	35.4
**CDK4/6 inhibitors**	1058	255	24.1
Palbociclib	776	167	21.5
Ribociclib	229	69	30.1
Abemaciclib	53	19	35.8
**VEGFR inhibitors**	1016	509	50.1
Sorafenib	191	100	52.4
Sunitinib	170	83	48.8
Cabozantinib	159	71	44.7
Other VEGFR inhibitors	496	255	51.4
**BCR-ABL inhibitors**	388	84	21.6
Imatinib	197	47	23.9
Dasatinib	79	17	21.5
Nilotinib	74	14	18.9
Other BCR-ABL inhibitors	38	6	15.8
**EGFR inhibitors**	325	129	39.7
Osimertinib	165	57	34.5
Afatinib	84	41	48.8
Erlotinib	49	27	55.1
Other EGFR inhibitors	27	4	14.8
**BRAF inhibitors**	250	109	43.6
Dabrafenib	182	84	46.2
Vemurafenib	38	12	31.6
Encorafenib	30	13	43.3
**MEK inhibitors**	241	110	45.6
Trametinib	186	88	47.3
Binimetinib	33	15	45.5
Cobimetinib	22	7	31.8
**ALK inhibitors**	153	61	39.9
Alectinib	71	16	22.5
Crizotinib	44	19	43.2
Lorlatinib	15	11	73.3
Other ALK inhibitors	23	15	65.2
**Other kinase inhibitors**	902	279	30.9
Ibrutinib	369	120	32.5
Ruxolitinib	265	60	22.6
Everolimus	174	69	39.7
Other kinase inhibitors	94	30	31.9
**HORMONE ANTAGONISTS**	1733	495	28.6
Abiraterone	1048	291	27.8
Enzalutamide	652	197	30.2
Apalutamide	33	7	21.2
**ANTI-NEOPLASTIC DRUGS**	1343	481	35.8
**Antimetabolites**	262	123	46.9
Trifluridine	256	122	47.7
Tegafur, gimeracil, oteracil	6	1	16.7
**PARP inhibitors**	220	51	23.2
Olaparib	128	35	27.3
Niraparib	87	14	16.1
Rucaparib	5	2	40.0
**Other anti-neoplastic drugs**	861	307	35.7
Temozolomide	465	168	36.1
Anagrelide	139	22	15.8
Venetoclax	117	69	59.0
Other anti-neoplastic drugs	140	48	34.3
**IMMUNOMODULATORS**	693	249	35.9
Lenalidomide	569	181	31.8
Pomalidomide	107	57	53.3
Thalidomide	17	11	64.7

* OAT sorted by descending numbers of first prescriptions. The three most frequently prescribed drugs per drug class are shown, and all other drugs are summarized in the category ‘others’. Abbreviations: CTCAE, Common Terminology Criteria for Adverse Events Abbreviations: ALK, anaplastic lymphoma kinase; BRAF, B-rapidly accelerated fibrosarcoma; CDK4/6, cyclin-dependent protein kinases 4/6; EGFR, epidermal growth factor receptor; MEK, mitogen-activated protein kinase; OAT, oral anticancer treatment; PARP, Poly(ADP-ribose) polymerase; VEGFR, vascular endothelial growth factor receptor.

**Table 4:** Types of adverse drug reactions, length of hospital stays, respective DRG data, and cost in the patients with OAT-related hospitalizations within AMBORA.

Patient Characteristics				G-DRG Data		
Patient ID	OAT	Type of Adverse Drug Reaction(s) Related to Hospitalization	Length of Stay (Days)	G-DRG Code	G-DRG Name	G-DRG cost (EUR)
**Intervention Group**						
1	Palbociclib	Blood count disorder	1	Q63B	Aplastic anemia	1176
2	Ibrutinib	Bleeding disorder	16	R61A	Lymphoma and non-acute leukemia with sepsis or a certain complicating constellation	17,024
3	Lenali-domide	Infection, blood count disorders	8	I66B	Other connective tissue disorders	7023
**Total**						**25,223**
**Control group**						
4 *	Niraparib	Gastrointestinal disorders	3	G67A	Esophagitis, gastroenteritis, gastrointestinal hemorrhage, ulcer disease, and various diseases of the digestive organs	2500
		Other disorders (hypokalemia)	2	L71Z	Renal failure	1621
5	Pazopanib	Other disorders (retinal detachment)	9	C03B	Interventions on the retina with pars plana vitrectomy, with extracapsular extraction of the lens (ECCE)	4008
6	Lenali-domide	Lung disorders	9	E74Z	Interstitial lung disease	2834
7 *	Lenvatinib	Cardiovascular disorder	2	F67D	Hypertension without a complicated diagnosis	1808
		Bleeding disorder	8	D13B	Small operations on the nose, ears, mouth and throat without complicating the diagnosis	2180
8	Tegafur, gimeracil, oteracil	Gastrointestinal disorders	9	G60B	Malignant growth of the digestive organs	2075
9	Everolimus	Infection, gastrointestinal disorder	2	G71Z	Other moderately severe diseases of the digestive organs	1990
10	Cabozan-tinib	Organ failure	9	H61A	Malignant neoplasm of the hepatobiliary system and pancreas	6409
11	Ribociclib	Blood count disorders	1	J62B	Malignant neoplasms of the breast	1491
12	Palbociclib	Blood count disorders	3	Q60C	Diseases of the reticuloendothelial system, immune system and coagulation disorders with complex diagnosis	2840
13	Ibrutinib	Organ failure, lung disorders	10	R03Z	Lymphoma and leukemia with a specific OR procedure	26,389
14	Venetoclax	Infection, blood count disorders, gastrointestinal disorders	12	R60C	Acute myeloid leukemia with int. chemotherapy	12,817
15	Midostaurin	Infection, blood count disorders, gastrointestinal disorders	9	R60E	Acute myeloid leukemia with moderately complex chemotherapy	5816
16	Everolimus	Infection, blood count disorder	2	T60F	Sepsis, died <5 days after admission	1951
17	Pazopanib	Cardiovascular disorder	1	X62Z	Poisoning/Toxic Effects of Drugs, Medicines and Other Substances	957
18	Pazopanib	Gastrointestinal disorders, other disorders (fatigue)	3	G67A	Esophagitis, gastroenteritis, gastrointestinal hemorrhage, ulcer disease, and various diseases of the digestive organs	2815
**Total**						**80,501**
**Mean per patient ± SD**			**6.0 ± 4.3**			**5873 ± 6612**

Abbreviations: G-DRG, German Diagnosis Related Groups; OAT, oral anticancer treatment; OR, operating room; SD, standard deviation. * Patients were hospitalized twice.

**Table 5:** Absolute risk for OAT-related hospitalizations derived from AMBORA and scenario analysis of patients in the SHI collective of AOK Bayern treated with new oral anticancer drugs.

	Number of Patients			Absolute Risk (%)
	OAT-Related Hospitalization	No OAT-Related Hospitalization	Total	
**AMBORA collective**				
**Intervention group** Intensified pharmacological/ pharmaceutical care program	3	95	98	3.06
**Control group** Standard of care	15	89	104	14.42
**SHI collective AOK Bayern**				
**Scenario 1** Standard of care for all patients	1025	6081	7106	14.42
**Scenario 2** Intensified pharmacological/pharmaceutical care program for all patients	217	6889	7106	3.06

Abbreviations: OAT, oral anticancer treatment; SHI, statutory health insurance.

## Data Availability

Not applicable.
